# Age of Rats Affects the Degree of Retinal Neuroinflammatory Response Induced by High Acute Intraocular Pressure

**DOI:** 10.1155/2022/9404977

**Published:** 2022-01-29

**Authors:** Shuhan Meng, Dan Wen, Jingge Xiao, Qianyue Zhang, Weizhou Fang, Xiao Xue, Tu Hu, Xiaobo Xia

**Affiliations:** ^1^Eye Center of Xiangya Hospital, Central South University, Changsha, Hunan, China 410008; ^2^Hunan Key Laboratory of Ophthalmology, Changsha, Hunan, China 410008; ^3^National Clinical Research Center for Geriatric Disorders, Xiangya Hospital, Central South University, Changsha, Hunan, China 410008; ^4^Xiangya Medical School, Central South University, Changsha, Hunan, China 410013

## Abstract

**Purpose:**

To investigate whether retinal neuroinflammatory response was affected by aging in a rat model of acute glaucoma.

**Methods:**

Young adult and aged rats were randomly assigned into normal control, 45 mmHg, 60 mmHg, and 90 mmHg groups. Intraocular pressure (IOP) of rats was acutely elevated to 45 mmHg, 60 mmHg, and 90 mmHg, respectively. Three days after high IOP treatment, loss of retinal ganglion cells (RGCs), formation of proinflammatory microglia/macrophages and neurotoxic astrocytes, and deposition of complement C3 in the retina were detected by immunofluorescence. ELISA was used to assess the protein levels of proinflammatory cytokines TNF and IL-1*β* in the retina.

**Results:**

Compared with young adult retinae, (1) loss of RGCs was more severe in aged retinae under the same IOP treatment, (2) microglia/macrophages were more prone to adopt proinflammatory phenotype in aged retinae in response to elevated IOP, (3) high IOP treatment induced astrogliosis, formation of neurotoxic astrocytes, and deposition of complement C3 more easily in aged retinae, and (4) aged retinae induced higher levels of proinflammatory cytokines TNF and IL-1*β* under the same IOP treatment.

**Conclusion:**

Our data indicated that aging affects the degree of retinal neuroinflammatory response initiated by ocular hypertension, which may contribute to the age-related susceptibility of RGCs to elevated IOP.

## 1. Introduction

Glaucoma is an age-related neurodegenerative disease and the leading cause of irreversible blindness worldwide [1]. It is marked by the degeneration of retinal ganglion cell (RGC) axons, soma, and synapses [2]. Clinical studies have clearly established that increased intraocular pressure (IOP) is the major risk factor in glaucoma [3]. By now, therapeutic strategy for all types of glaucoma is limited to the reduction of elevated IOP, which does not completely stop the progression of glaucomatous neurodegeneration and visual field defects [4]. Besides IOP, age is another main risk factor of glaucoma [5, 6]. A body of studies has demonstrated that the prevalence of glaucoma increases markedly with advancing age across all populations [1, 7, 8].

Consistent with these findings, we previously identified the existence of the age-related susceptibility of rat retinae to increased IOP [9]. Other reports suggested that age-related mitochondria dysfunction and accumulation of oxidative damage make RGCs more vulnerable to damage in the progression of glaucomatous neurodegeneration [10, 11]. Moreover, chronic exposure of tissue stress (oxidized lipoproteins and free radicals) triggered the low-grade inflammation (parainflammation) in aged retinae [12]. A physiological level of parainflammation was critical for maintaining the retinal homeostasis, but when injuries occurred, dysregulated parainflammation developed into detrimental inflammatory response which exacerbates the glaucomatous neurodegeneration [13]. Similar to other neurodegenerative diseases, resident microglia and astrocytes as well as infiltrated macrophages participated in the retinal neuroinflammatory response in glaucoma [14]. These immune cells could recognize and respond to various stimuli including glaucoma-related stress and damage-associated molecular patterns (DAMPs) arising from stressed or damaged RGCs [15]. Recent studies have well established that microglia/macrophages and astrocytes can be activated by these stimuli and transform into the proinflammatory/neurotoxic phenotype [16–19]. In glaucoma, these proinflammatory microglia/macrophages and neurotoxic astrocytes produced proinflammatory cytokines (e.g., tumor necrosis factor (TNF), interleukin-1*β* (IL-1*β*), IL-6, and IL-12), chemokines (e.g., C-C motif chemokine ligand 2 (CCL2), C-X-C motif chemokine ligand 2 (CXCL2), and CXCL10), complement component (e.g., C1q, C3, and C4), reactive oxygen species (ROS), and nitric oxide (NO) to directly induce RGC death or amplify the retinal neuroinflammatory response [16].

Recent evidence hinted that some microglia and astrocytes lose their ability to maintain homeostasis and adopt a more proinflammatory or “primed” phenotype as a result of the aging process [20–22]. Compared to the resting microglia and astrocytes, these “primed” microglia and astrocytes were more sensitive to inflammatory cues and displayed the more robust inflammatory response, as the higher expression of proinflammatory cytokines/chemokines and complement component [23, 24]. Our previous results showed that high IOP treatment induces the more evident activation of microglia/macrophages (characterized by the more IBA1- (ionized calcium-binding adapter molecule 1-) positive cells and IBA1-positive stained area) in aged retinae than young adult retinae [9]. However, the proinflammatory features of microglia/macrophages have not been identified in this scenario.

In the present study, we detected the formation of proinflammatory microglia/macrophages and neurotoxic astrocytes, deposition of complement C3, and production of proinflammatory cytokines (TNF and IL-1*β*) in the rat glaucoma model we previously used [9]. These findings raised the possibility that retinal neuroinflammatory response may contribute to the age-related vulnerability of RGCs in glaucoma.

## 2. Materials and Methods

### 2.1. Animal and Grouping

Thirty-six young adult (aged 2 months, 200–250 g) and thirty-six aged (aged 15 months, 300-350 g) female Sprague-Dawley rats were supplied from Central South University, Changsha, Hunan Province, People's Republic of China. All rats were housed under controlled environmental conditions on a 12 h light/dark cycle with ad libitum access to food and water. All experiments were approved by the animal ethics committee in the Xiangya Hospital of Central South University. Young adult and aged rats were randomly divided into normal control (*n* = 9; *n* = 3, 6 eyes for retinal whole mounts; *n* = 3, 6 eyes for retinal cross-sections; and *n* = 3, 6 eyes for ELISA test), 45 mmHg (*n* = 9; *n* = 3, 6 eyes for retinal whole mounts; *n* = 3, 6 eyes for retinal cross-sections; and *n* = 3, 6 eyes for ELISA test), 60 mmHg (*n* = 9; *n* = 3, 6 eyes for retinal whole mounts; *n* = 3, 6 eyes for retinal cross-sections; and *n* = 3, 6 eyes for ELISA test), and 90 mmHg (*n* = 9; *n* = 3, 6 eyes for retinal whole mounts; *n* = 3, 6 eyes for retinal cross-sections; and *n* = 3, 6 eyes for ELISA test) groups. IOP of rats in 45 mmHg, 60 mmHg, and 90 mmHg groups was increased acutely to 45 mmHg, 60 mmHg, and 90 mmHg, respectively. All rats were sacrificed with an i.p. overdose of pentobarbital at 3 days after high IOP treatment.

### 2.2. Acute IOP Model

The animal model was prepared following the reported method [9, 25]. Briefly, under anesthesia of 2% pentobarbital (40 mg/kg), a 30-gauge infusion needle connected to the installation instrument filled with sterile saline was inserted into the anterior chamber of rats. The IOP was raised to 45 mmHg, 60 mmHg, or 90 mmHg for 60 min. After maintenance of 60 min, the 90 mmHg of IOP was decreased through 80 mmHg for 5 min, 70 mmHg for 5 min, 60 mmHg for 5 min, and 30 mmHg for 5 min. For the condition of 45 mmHg and 60 mmHg of IOP, the IOP was directly decreased to 30 mmHg after maintenance of 60 min.

### 2.3. Retinal Tissue Preparation

After anesthetizing, rats were transcardially perfused with ice-cold 0.9% saline followed by ice-cold 4% paraformaldehyde. The eyeballs were enucleated, and the corneas, lenses, and vitreous bodies were removed. For retinal cross-sections, the remaining eye cups were postfixed overnight in 4% paraformaldehyde at 4°C, placed into 30% sucrose for cryoprotection, and embedded in OCT compound (Tissue-Tek). The 20 *μ*m tissue sections were prepared with a Leica cryostat. For retinal whole mounts, the remaining eye cups were postfixed in 4% paraformaldehyde for 1 h, and the whole retinas were dissected and processed as described previously [26].

For ELISA, rats were transcardiac perfused with ice-cold 0.9% saline. The eyeballs were enucleated and retinae were collected. Then retinae were homogenized by sonication on ice in a digestion buffer containing protease inhibitors (Sigma). The homogenates were treated with centrifugation, protein concentration determination, and denaturation.

### 2.4. Immunofluorescence

Retinal whole mounts and cross-sections were blocked with PBS containing 5% BSA and 0.3% Triton X-100 for 1 h at room temperature and incubated with primary antibodies diluted in PBS containing 5% BSA and 0.1% Triton X-100 overnight at 4°C (rabbit anti-rat RBPMS (RNA-binding protein with multiple splicing), GeneTex, GTX118619, 1 : 500; rabbit anti-rat IBA1, Wako, 019-19741, 1 : 200; mouse anti-rat CD68, Abcam, ab31630, 1 : 200; mouse anti-rat MHC-II (major histocompatibility complex class II), Abcam, ab23990, 1 : 200; mouse anti-rat GFAP (glial fibrillary acidic protein), Cell Signaling Technology, #3670, 1 : 2000; and rabbit anti-rat C3, Abcam, ab200999, 1 : 200). Then the retinal whole mounts and cross-sections were incubated with secondary antibodies labeled with fluorescent dyes (1 : 1000, Jackson ImmunoResearch) for 2 h at room temperature followed by nuclei staining with DAPI (Vector Laboratories). As negative controls, an adjacent series of sections were processed in parallel without the primary antibodies.

### 2.5. Enzyme-Linked Immunosorbent Assay Analysis (ELISA)

The levels of secreted TNF and IL-1*β* in the retinal supernatants were detected by rat TNF (#CSB-E11987r, CUSABIO, China) and IL-1*β* (#CSB-E08055r, CUSABIO, China) ELISA kits. Briefly, the testing sample and standard sample were added in duplicates to plates precoated with the primary antibody and incubated for 2 h at 37°C. Next, the biotin-labeled secondary antibody was added and incubated for 1 h at 37°C. After incubation, HRP-conjugate reagent was added followed by repeat incubation. Then, tetramethylbenzidine enzyme-substrate (TMB) was added and incubated for 30 min at 37°C. Finally, stop solution was added for stopping the reaction, and the absorbance (OD450) of all BSA Standards and samples were recorded within 5 min.

### 2.6. Cell Counting and Data Analysis

The central and peripheral regions of the rat retina were defined as previously described [27]. For each retinal whole mount, twelve images from the central and peripheral region of the retina were taken under 20x magnification, respectively, using the Leica DM6000 fluorescence microscope. RBPMS-labeled RGCs in each 20x field were counted. A total RBPMS^+^ cell number of 12 fields per region were averaged to represent RGC density in the central and peripheral retinae, respectively.

Three sections from each eye for CD68/IBA1, MHC-II/IBA1, and GFAP/C3 double staining were chosen. The proinflammatory microglia/macrophages were defined by colabeling of IBA1 (microglia/macrophages marker) and CD68 (phagocytic marker)/MHC-II (antigen presenting molecular). The neurotoxic astrocytes were defined by colabeling of GFAP (astrocyte marker) and complement C3. Two images from the central region for each retinal cross-section were randomly captured under 40x magnification using the Zeiss LSM780 confocal microscope. The number of CD68^+^IBA1^+^ and MHC-II^+^IBA1^+^ cells in captured images of the inner retina (including nerve fiber layer, ganglion cell layer, and inner plexiform layer) was blindly counted, respectively. Relative mean gray value and relative positive area of GFAP and C3 staining in the inner retina were measured by ImageJ.

All data were presented as mean ± standard deviation (mean ± SD). Two-way analysis of variance (ANOVA) followed by Tukey's multiple comparisons test was used to assess the statistical significance. Assessments with *p* < 0.05 were considered significant.

## 3. Results

### 3.1. The Same High IOP Caused More Serious Loss of RGCs in Aged Retinae than in Young Adult Retinae

**Figure 1:**
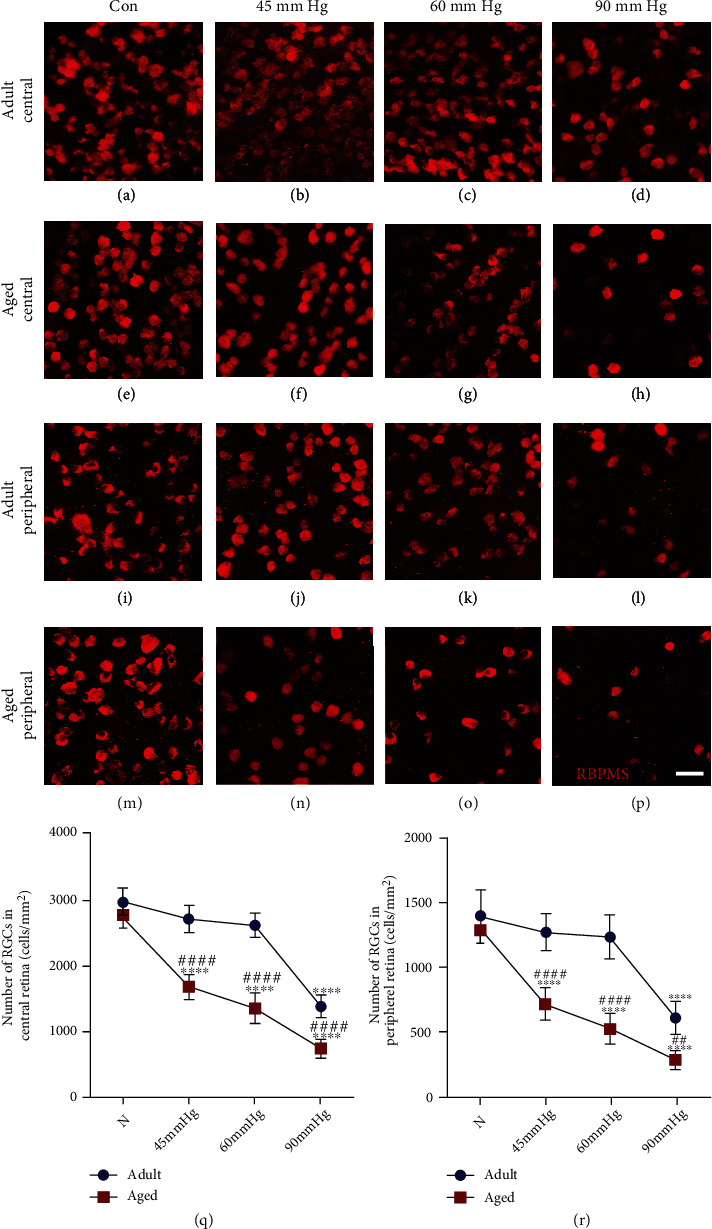
RGCs of aged retinae were more vulnerable to increased IOP than that of young adult retinae. Compared with the age-matched normal control (i, m), IOP treatment of 90 mmHg was sufficient to induce significant loss of RGCs in central (d, q) and peripheral regions (l, r) of young adult retina 3 days after treatment. Since IOP of 45 mmHg, the aged retina showed significant loss of RGCs in central (f–h, q) and peripheral regions (n–p, r). RGC loss in aged retinae was significantly higher than that of young adult retinae at each IOP treatment (q, r) (^∗∗∗∗^*p* < 0.0001 versus age-matched control; ^##^*p* < 0.01, ^####^*p* < 0.0001 versus matched part of young adult retina at the same IOP treatment. Scale bar: 100 *μ*m).

Loss of RGCs is the major pathological hallmark of glaucoma [27]. Here, we assessed the RGC loss by counting the number of RBPMS- (a specific marker of RGC in the retina) labeled cells in retinal whole mounts of aged and young adult rats 3 days after IOP treatment of 45 mmHg, 60 mmHg, and 90 mmHg (Figure 1). Two-way ANOVA analysis showed that aging exacerbates the loss of RGCs after IOP treatment (*F*(1, 40) = 198.97, *p* < 0.0001, for central; *F*(1, 40) = 110.26, *p* < 0.001, for peripheral). Moreover, increased IOP also exerted effects on RGC loss (*F*(3, 40) = 181.65, *p* < 0.0001, for central; *F*(3, 40) = 84.70, *p* < 0.001, for peripheral). Compared with the normal aged retinae (Figures 1(e) and 1(m)), the number of RGCs in central and peripheral regions of aged retinae was significant decreased since IOP of 45 mmHg compared with the normal aged retinae (Figures 1(f), 1(h), 1(q), 1(n)–1(p), and 1(r)). However, significant loss of RGCs in central and peripheral retinae of young adult rats could not be detected until at 90 mmHg (Figures 1(d), 1(q), 1(l), and 1(r)). Of note, RGC loss was more evident in aged retinae than young adult retinae at each IOP treatment (Figures 1(q) and 1(r)). These results were consistent with our previous data [9], indicating that aged retinae are more vulnerable to increased IOP compared to young adult retinae.

### 3.2. Microglia/Macrophages of Aged Retinae Were More Prone to Exhibit Proinflammatory Phenotype than That of Young Adult Retinae after High IOP Treatment

**Figure 2:**
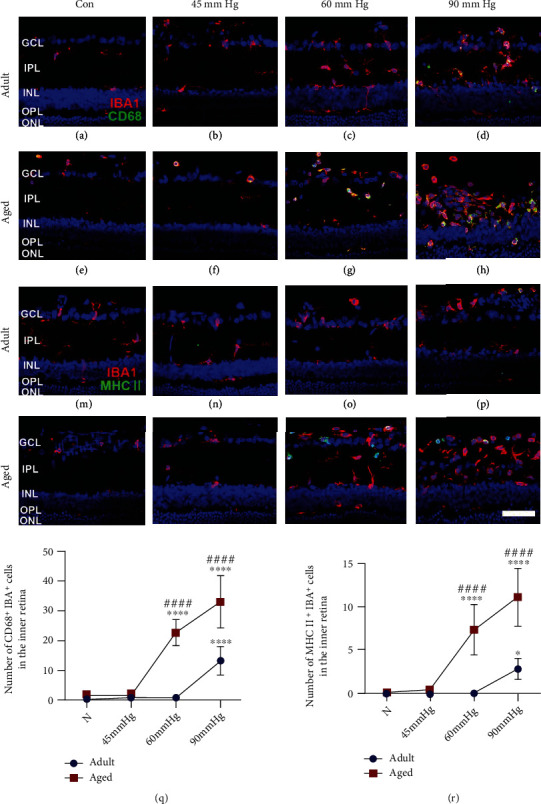
Microglia/macrophages were more prone to exhibit proinflammatory phenotype in aged retinae than that of young adult retinae after IOP treatment. Significant increase in CD68^+^IBA1^+^ (d, q) and MHC-II^+^IBA1^+^ cells (l, r) was observed in young adult retinae at IOP of 90 mmHg. Aged retinae showed dramatic increase in CD68^+^IBA1^+^ (g–h, q) and MHC-II^+^IBA1^+^ cells (o–p, r) since IOP of 60 mmHg. Compared with the young adult retinae, aged retinae induced more CD68^+^IBA1^+^ (q) and MHC-II^+^IBA1^+^ cells (r) at 60 and 90 mmHg 3 days after IOP treatment (GCL: ganglion cell layer; IPL: inner plexiform layer; INL: inner nuclear layer; OPL: outer plexiform layer; ONL: outer nuclear layer. ^∗^*p* < 0.05, ^∗∗∗∗^*p* < 0.0001 versus age-matched control; ^####^*p* < 0.0001 versus matched part of young adult retina at the same IOP treatment. Scale bar: 50 *μ*m).

In glaucoma, activated microglia/macrophages could adopt proinflammatory phenotype that contributes to neuronal death by releasing neurotoxic factors [28]. These proinflammatory microglia/macrophages might upregulate CD68, a lysosomal marker indicative of phagocytic activity of microglia/macrophages, and antigen presentation molecule MHC-II [16]. Here, we detected the proinflammatory microglia/macrophages in the inner retina by double immunostaining of IBA1 and CD68/MHC-II (Figure 2). Two-way ANOVA analysis showed that aging significantly increases the number of CD68^+^IBA1^+^ and MHC-II^+^IBA1^+^ cells 3 days after IOP treatment (*F*(1, 40) = 116.72, *p* < 0.001, for CD68^+^IBA1^+^ cells; *F*(1, 40) = 156.89, *p* < 0.001, for MHC-II^+^IBA1^+^ cells). IOP treatment also significantly increased the number of CD68^+^IBA1^+^ and MHC-II^+^IBA1^+^ cells (*F*(3, 40) = 250.13, *p* < 0.001, for CD68^+^IBA1^+^ cells; *F*(3, 40) = 190.03, *p* < 0.001, for MHC-II^+^IBA1^+^ cells). The significant increases in CD68^+^IBA1^+^ and MHC-II^+^IBA1^+^ cells were detected in aged retinae since IOP of 60 mmHg (Figures 2(g), 2(h), 2(q), 2(o), 2(p), and 2(r)) and in young adult retinae at 90 mmHg (Figures 2(d), 2(q), 2(l), and 2(r)). The number of CD68^+^IBA1^+^ and MHC-II^+^IBA1^+^ cells in aged retinae was significantly higher than that of young adult retina at 60 and 90 mmHg (Figures 2(q) and 2(r)). These findings suggested that increased IOP induces the proinflammatory activation of microglia/macrophages more easily in aged retinae than young adult retinae.

### 3.3. Aged Retinae Were More Prone to Induce Astrogliosis and C3 Deposition than Young Adult Retinae in Response to Increased IOP

**Figure 3:**
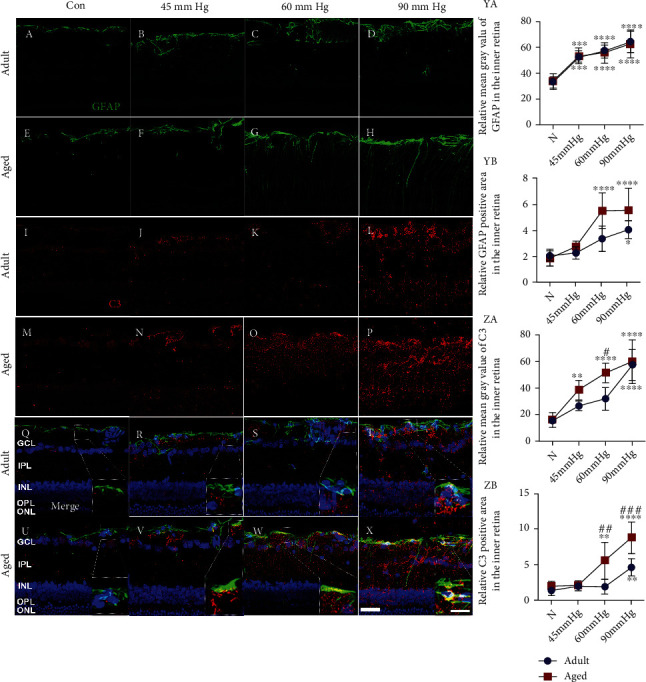
High IOP treatment induced astrogliosis and C3 deposition more easily in aged retinae than in young adult retinae. High IOP treatment induced the increase in GFAP and C3 staining in young adult (a–d, i–l) and aged retinae (e–h, m–p). Merged images showed colocalization (yellow) of C3 and GFAP staining, suggesting the formation of neurotoxic astrocytes in young adult (q–t) and aged retinae (u–x) 3 days after treatment. Quantification of GFAP and C3 expression in the inner retina was presented as the relative mean gray value and relative positive area (YA-YB, ZA-ZB) (GCL: ganglion cell layer; IPL: inner plexiform layer; INL: inner nuclear layer; OPL: outer plexiform layer; ONL: outer nuclear layer. ^∗^*p* < 0.05, ^∗∗^*p* < 0.01, ^∗∗∗^*p* < 0.001, ^∗∗∗∗^*p* < 0.0001 versus age-matched control; ^#^*p* < 0.05, ^##^*p* < 0.01, ^####^*p* < 0.0001 versus matched part of young adult retina at the same IOP treatment. Scale bar: 50 *μ*m, zoom: 20 *μ*m).

We previously found that no significant difference of astrogliosis (quantified by the relative mean gray value of reactive astrocyte marker GFAP) exists between young adult and aged retinae under the same high IOP treatment [9]. In the present study, we assessed the astrogliosis represented as the relative mean gray value of GFAP and relative GFAP^+^ area in the inner retinae (Figure 3). Two-way ANOVA analysis showed that IOP treatment significantly increases the relative mean gray value of GFAP (*F*(3, 40) = 39.64, *p* < 0.0001). However, aging shows no effects on the relative mean gray value of GFAP in the inner retina after IOP treatment (*F*(1, 40) = 0.13, *p* = 0.7213) (Figure 3). Of note, we found that aging affects the positive staining area of GFAP after IOP treatment (*F*(1, 40) = 13.61, *p* = 0.0007). Moreover, IOP treatment significantly increased the GFAP^+^ area in the inner retinae (*F*(3, 40) = 27.18, *p* < 0.0001). Significant increase in the GFAP^+^ area in the young adult retinae was not detected until at IOP of 90 mmHg (Figures 3(a)–3(d), YB). The GFAP^+^ area in aged retinae was increased since IOP of 60 mmHg (Figures 3(e)–3(h), YB). Additionally, the GFAP^+^ area in aged retinae was significantly higher than young adult retinae at 60 mmHg (Figure 3, YB). These results suggested that strong astrogliosis is induced more easily in aged retinae than young adult retinae.

Reactive astrocytes might adopt neurotoxic phenotype in glaucoma to drive neurodegenerative process [29]. These neurotoxic astrocytes are marked by the production of complement C3 [30]. Recent studies identified that retinal deposition of C3 contributes to glaucomatous neurodegeneration [31]. We saw the upregulation of C3 within astrocytes in both aged and young adult retinae in response to elevated IOP (Figures 3(q)–3(t) and 3(u)–3(x)). Further, we assessed the C3 deposition in the inner retinae by measurement of the relative mean gray value of C3 and C3^+^ areas (Figure 3, ZA and ZB). We found that aging affects the relative mean gray value (*F*(1, 40) = 11.05, *p* = 0.0019) and positive staining area (*F*(1, 40) = 28.24, *p* < 0.0001) of C3. In addition, IOP treatment dramatically increased the relative mean gray value (*F*(3, 40) = 47.58, *p* < 0.0001) and positive area (*F*(3, 40) = 33.37, *p* < 0.0001) of C3. We found that the relative mean gray value and positive area of C3 in aged retinae are not different from that of young adult retinae at the normal control (Figures 3(i) and 3(m), ZA and ZB). The increase in the relative mean gray value of C3 was detected in aged retinae since IOP of 45 mmHg, whereas the C3^+^ area was increased at 60 and 90 mmHg (Figures 3(n)–3(p), ZA and ZB). However, significant increase in the relative mean gray value and C3^+^ area could not be detected in young adult retinae until at IOP of 90 mmHg (Figure 3(l), ZA and ZB). Moreover, aged retinae showed significantly higher levels of the relative mean gray value of the C3 at 60 mmHg and C3^+^ area at 60 and 90 mmHg than young adult retinae (Figure 3, ZA and ZB). These results suggested that reactive astrocytes may be more primed to adopt neurotoxic phenotype in aged retinae than in young adult retinae, which is characterized by the higher levels of C3 deposition in the inner part of retinae.

### 3.4. High IOP Treatment Induced Higher Production of Proinflammatory Cytokines in Aged Retinae than in Young Adult Retinae

**Figure 4:**
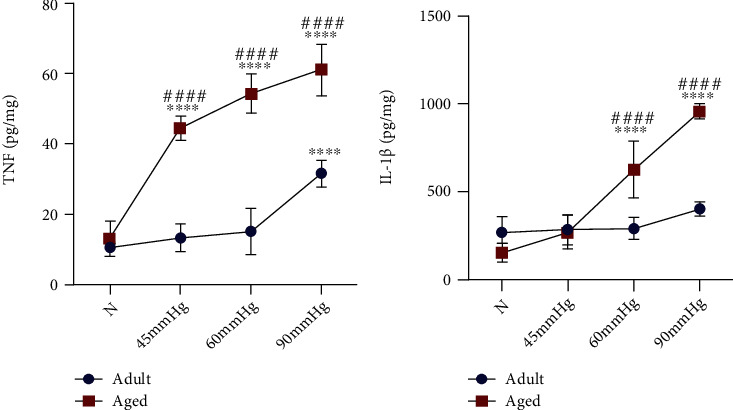
Aged retinae induced higher production of proinflammatory cytokines than young adult retinae after high IOP treatment. TNF levels were significantly increased in young adult retinae at IOP of 90 mmHg and in aged retinae since IOP of 45 mmHg (a). The protein levels of TNF in aged retinae were significantly higher than in young adult retinae at each IOP treatment (a). IL-1*β* levels were dramatically increased in aged retinae since IOP of 60 mmHg, while no significant increase in IL-1*β* was detected in young adult retinae after IOP treatment (b). IL-1*β* production in aged retinae was significantly higher than young adult retinae since IOP of 60 mmHg (b) (^∗∗∗∗^*p* < 0.0001 versus age-matched control; ^####^*p* < 0.0001 versus matched part of young adult retina at the same IOP treatment).

Proinflammatory microglia/macrophages and neurotoxic astrocytes produced various proinflammatory cytokines such as TNF and IL-1*β* to mediate neuroinflammatory response [28, 32]. Here, we detected the protein levels of TNF and IL-1*β* in rat retinae by ELISA (Figures 4(a) and 4(b)). Two-way ANOVA results suggested that aging seriously affects the TNF and IL-1*β* protein levels after IOP treatment (*F*(1, 40) = 320.48, *p* < 0.0001, for TNF; *F*(1, 40) = 60.71, *p* < 0.0001, for IL-1*β*). Moreover, IOP treatment significantly increased the TNF and IL-1*β* protein levels (*F*(3, 40) = 100.43, *p* < 0.0001, for TNF; *F*(3, 40) = 73.58, *p* < 0.0001, for IL-1*β*). We saw that TNF protein levels are dramatically increased in aged retinae since IOP of 45 mmHg (Figure 4(a)). The increase in TNF protein levels in young adult retinae could not be detected until at IOP of 90 mmHg (Figure 4(a)). The protein levels of IL-1*β* in aged retinae were significantly elevated since IOP of 60 mmHg (Figure 4(b)). Young adult retinae showed modest but not statistically significant increase in IL-1*β* levels at 90 mmHg (*p* = 0.3323) (Figure 4(b)). The protein levels of TNF in aged retinae were significantly higher than that of young adult retinae at each IOP treatment (Figure 4(a)). Aged retinae also showed higher degree of IL-1*β* protein levels than that of young adult retinae since IOP of 60 mmHg (Figure 4(b)). Collectively, these data indicated that high IOP treatment induces stronger neuroinflammatory response in aged retinae compared to young adult retinae.

## 4. Discussion

The purpose of this study was to investigate if age of rats affects the retinal neuroinflammatory response in acute rat glaucoma. Here, we demonstrated that compared to young adult retinae, (1) RGCs in aged retinae are more susceptible to acute IOP elevation, (2) microglia/macrophages are more prone to adopt the proinflammatory phenotype in response to IOP elevation in aged retinae, (3) astrogliosis and C3 deposition are induced more easily in aged retinae, and (4) high IOP treatment induces higher levels of proinflammatory cytokines in aged retinae. These results supported that aged retinae induce more severe neuroinflammatory response than young adult retinae in acute glaucoma.

Loss of RGCs was the main pathological hallmark of glaucomatous degeneration [33]. In our previous study, loss of neurons at ganglion cell layer (GCL) after high IOP treatment was measured by counting the number of NeuN^+^ cells in retinal cross-sections. Here, we quantified the loss of RGCs by counting RBPMS^+^ (a specific marker of RGC in retina) cells in retinal whole mounts. The results of RGC loss in this study were consistent with the findings we have previously reported [9], which indicates the existence of age-related susceptibility of RGC to elevated IOP.

In glaucoma, microglia/macrophages might transform into “ameboid” morphology and upregulate phagocytosis-associated protein CD68, antigen presentation molecule MHC-II, and other neurotoxic molecules, displaying the proinflammatory and phagocytic phenotypes [34, 35]. In the present study, we saw the upregulation of CD68 and MHC-II in IBA1^+^ microglia/macrophages of the retina after high IOP treatment. We found that significant increase in CD68^+^IBA1^+^ and MHC-II^+^IBA1^+^ cells could not be detected in young adult retinae until IOP of 90 mmHg. However, aged retinae increased CD68^+^IBA1^+^ and MHC-II^+^IBA1^+^ cells at 60 mmHg and more as IOP increased. Moreover, the number of CD68^+^IBA1^+^ and MHC-II^+^IBA1^+^ cells in aged retinae was significantly higher than young adult retina at 60 and 90 mmHg. These findings indicated that microglia/macrophages are more prone to adopt proinflammatory phenotype in aged retinae in response to elevated IOP. Recent studies discovered several factors contribute to the age-related proinflammatory changes in retinal microglia, including the altered metabolism and reduced immunoregulatory signaling (e.g., CX3CL1-CX3CR1 and CD200-CD200R signaling) from the retinal neurons [11]. In addition, Tang et al. unraveled that epigenetic modification may induce the aged-related proinflammatory alterations in microglia [36]. In their reports, decreased expression of histone demethylases Jumonji domain-containing protein 3 (Jmjd3) and increased levels of Tri-methylation lysine 27 of histone H3 (H3K27me3) were detected in the midbrain of aged mice, which is accompanied by the upregulation of proinflammatory microglia markers (TNF, IL-6, and nitric oxide synthase 2 (NOS2)) and downregulation of anti-inflammatory microglia marker Arginase-1 (Arg-1) [36].

Recent studies identified that neurotoxic astrocytes were major effectors to drive glaucomatous neurodegeneration [29, 37]. However, the detailed mechanisms underlying how these neurotoxic astrocytes contribute to the damage of retinal neurons remained unclear. As the specific marker of neurotoxic astrocytes, complement C3 was tightly associated with neuroinflammation [30]. Lian et al. found that astrocyte-released C3 impaired dendritic morphology and network functions of cortical neurons through neuronal C3aR (C3a receptor) [38]. On the other hand, astrocyte-derived C3 could induce the proinflammatory activation of microglia via triggering the microglial C3aR [39]. In the present study, we firstly detected the degree of astrogliosis by immunostaining of reactive astrocyte marker GFAP. We found that high IOP treatment induces the increase in GFAP^+^ area more easily in aged retinae than in young adult retinae. The obvious GFAP-positive processes in the inner plexiform layer were observed in aged retinae at IOP of 60 mmHg, while the GFAP^+^ area in young adult retinae was limited to the ganglion cell layer under the same IOP. These findings implied that astrocytes in aged retinae may be more sensitive to increased IOP.

Next, we found upregulation of C3 within astrocytes in both young adult and aged retinae in response to increased IOP, suggesting the formation of neurotoxic astrocytes. Recent studies have identified that C3 contributes to glaucomatous neurodegeneration [31]. Here, we showed higher degree of C3 deposition in aged retinae than in young adult retinae since IOP of 60 mmHg. These findings might provide evidence for the existence of age-related proinflammatory changes in retinal astrocytes. In fact, recent studies unraveled that normal aging can induce the proinflammatory changes of astrocytes in the mouse brain, which is characterized by the upregulation of genes involved in complement, inflammatory cytokines, and antigen presentation pathways [24]. Of note, it was identified that aging-induced proinflammatory changes of astrocytes is mediated by microglia-released inflammatory signal (TNF, IL-1*β*, and C1q) factors. This indicated that aging-induced alterations of microglia and astrocytes may be a coordinative process [24].

Proinflammatory microglia/macrophages and neurotoxic astrocytes were the main source of proinflammatory cytokines such as TNF and IL-1*β* in the context of glaucoma [40]. Several studies revealed that TNF/TNF receptor 1 (TNFR1) signaling can directly induce RGC death via proteolytic caspase cascade, mitochondrial dysfunction, and oxidative damage [41–43]. On the other hand, TNF and IL-1*β* could promote the proinflammatory activation of microglia/macrophages and astrocytes in the autocrine or amacrine manner, which further amplifies the neuroinflammatory response [44]. Thus, we detected the TNF and IL-1*β* protein levels to evaluate the degree of retinal neuroinflammatory response after different IOP treatments. We found that significant increase in TNF protein levels cannot be detected in young adult retinae until IOP of 90 mmHg. In addition, young adult retinae showed a slight but not statistically significant increase in IL-1*β* at 90 mmHg. However, aged retinae upregulate TNF and IL-1*β* levels at IOP of from 45 to 90 mmHg. Besides, upregulation of TNF and IL-1*β* in aged retinae was significantly higher than that of young adult retina under the same IOP. These findings indicated that elevated IOP might induce a more severe neuroinflammatory response in aged retinae than young adult retinae.

Taken together, our findings highlighted that aging can seriously affect the degree of retinal neuroinflammatory response in acute rat glaucoma. This might explain the age-related vulnerability of RGCs to increased IOP. Further studies should identify the molecular mechanisms that drive the age-related proinflammatory changes within microglia/macrophages and astrocytes, which may provide the promising target for glaucoma treatment.

## Data Availability

The data used to support the findings of this study are available from the corresponding author upon request.
